# Deficiency of IL-27 Signaling Exacerbates Experimental Autoimmune Uveitis with Elevated Uveitogenic Th1 and Th17 Responses

**DOI:** 10.3390/ijms22147517

**Published:** 2021-07-14

**Authors:** Sihan Wu, Rui Ma, Yajie Zhong, Zilin Chen, Hongyan Zhou, Minyi Zhou, Waipo Chong, Jun Chen

**Affiliations:** State Key Laboratory of Ophthalmology, Zhongshan Ophthalmic Center, Sun Yat-Sen University, Guangzhou 510060, China; wush55@mail2.sysu.edu.cn (S.W.); marui8@mail2.sysu.edu.cn (R.M.); zhongyj27@mail2.sysu.edu.cn (Y.Z.); chenzil3@mail2.sysu.edu.cn (Z.C.); zhouhongyan@gzzoc.com (H.Z.); zhouminyi@gzzoc.com (M.Z.)

**Keywords:** EAU, IL-27, GM-CSF, Th1, Th17, Tr1

## Abstract

Human uveitis is an autoimmune disease of the central nervous system that is characterized by ocular inflammation with the involvement of uveitogenic Th1 and Th17 responses. In experimental autoimmune uveitis (EAU), the animal model for human uveitis, both responses are proven to be critical in disease development. Therefore, targeting both Th1 and Th17 cells has therapeutic implication for disease resolution. IL-27 is a multifunctional cytokine that can either promote or inhibit T cell responses and is implicated in both autoimmune and infectious diseases. The aim of this study is to characterize the role of IL-27/IL-27R signaling in regulating uveitogenic Th1/Th17 responses in EAU. By immunizing IL-27Rα^−/−^ mice and their wild-type (WT) littermates for EAU, we demonstrated that IL-27 signaling deficiency exacerbated EAU with severe ocular inflammation and impairment of visual function. Furthermore, there was a significant increase in the eye-infiltrating Th1 and Th17 cells in IL-27Rα^−/−^ EAU mice compared to WT. Their retinal antigen-specific Th1 and Th17 responses were also significantly increased, as represented by the elevation of their signature cytokines, IFN-γ and IL-17A, respectively. We also observed the upregulation of another pathogenic cytokine, granulocyte-macrophage colony-stimulating factor (GM-CSF), from effector T cells in IL-27Rα^−/−^ EAU mice. Mechanistic studies confirmed that IL-27 inhibited GM-CSF production from Th17 cells. In addition, the induction of IL-10 producing type 1 regulatory T (Tr1) cells was impaired in IL-27Rα^−/−^ EAU mice. These results identified that IL-27 signaling plays a suppressive role in EAU by regulating multiple CD4^+^ cell subsets, including the effector Th1 and Th17 cells and the regulatory Tr1 cells. Our findings provide new insights for therapeutic potential in controlling uveitis by enhancing IL-27 signaling.

## 1. Introduction

Uveitis is an intraocular inflammatory disease that affect the retina, optic nerve, and uvea tract, which includes iris, ciliary body, and choroid. This disease is one of the leading causes of vision impairment in the developed world and is responsible for 10–15% of severe visual handicap [1]. Uveitis is characterized by chronic and recurrent ocular inflammation that lasts for years [2]. Many cases have been diagnosed among working-age population, causing significant socioeconomic burden and public health issues.

Experimental autoimmune uveitis (EAU) is a “classical” animal model of human uveitis [3,4]. It mimics clinical manifestations of human disease, including retinal vasculitis/retinitis, choroidal inflammation, and photoreceptor destruction [5]. Previous studies on EAU have demonstrated that autoreactive CD4^+^ T helper (Th) cells are the major cause of inflammation, leading to blood–retinal barrier breakdown and cellular infiltration into the immunologically privileged sites, the eyes [6].

Among the CD4^+^ T cell subsets, Th1 and Th17 cells play a critical role in ocular inflammation and autoimmune eye diseases [7,8], and either of them could drive the disease pathogenesis independently in EAU [9]. Elevated Th1 and Th17 responses are associated with clinically definite uveitis in patients [10,11]. Th1 and Th17 cells are derived from a common pool of antigen-specific precursors, which can be differentiated along either pathway. Previous observations in animal models further prove that neutralization or deficiency of one response promotes the alternative response [12]. For example, although Th1 cells and their signature cytokine IFN-γ are demonstrated to be pathogenic in EAU [13], innate IFN-γ from natural killer (NK) cells and natural killer T (NKT) cells are protective, as systemic depletion of IFN-γ exacerbates the disease development, in association with elevated Th17 response, in EAU [14,15] and experimental autoimmune encephalomyelitis (EAE), an animal model for multiple sclerosis [16]. Additionally, IL-23-driven autoreactive Th17 cells have been identified as the dominant factor for the disease pathogenesis in autoimmune diseases, including uveitis, multiple sclerosis, arthritis, psoriasis, and inflammatory bowel disease [17], while depletion of IL-17A in Th17 cells do not reduced their pathology in neuroinflammation [18]. Therefore, we proposed that the ideal therapy for uveitis should target both Th1 and Th17 lineages concurrently [19].

IL-27, one of the IL-12 family members, is a heterodimeric cytokine composed of IL-27p28 and EBI3. It primarily produced by activated antigen-presenting cells (APCs), and some other cell types including astrocytes and microglia [20,21]. The IL-27 receptor is a heterodimer composed of IL-27Rα (also called TCCR and WSX-1) and gp130. IL-27Rα is unique for the cytokine and is widely expressed on numerous cell types, including CD4^+^ T cells, NK cells, and NKT cells, but gp130 is shared by other cytokine receptors such as IL-6 and IL-35 [22]. Functionally, the binding of IL-27 and its receptor IL-27R is essential for early priming of Th1 response [23]. To study the role of IL-27Rα in vivo, researchers generated IL-27Rα^−/−^ mice, which displayed normal development of hematopoietic and lymphoid systems when compare to the WT mice [23]. However, recent studies revealed that IL-27/IL-27R signaling is essential for immunocontraction: (1) IL-27Rα-deficient NK, NKT cells, and CD4^+^ T cells produce more pro-inflammatory cytokines, such as IFN-γ and IL-17A [14,24]; (2) IL-27 inhibits Th17 response directly through downregulation of RORγt [25]; (3) indirectly suppresses both Th1 and Th17 response by induction of IL-10-producing type 1 regulatory T (Tr1) cells [26]; and (4) restrains the production of the proinflammatory cytokine GM-CSF in neuroinflammation [27,28]. Clinical studies illustrated that lower IL-27 expression level is associated with the Th17 response in patients with uveitis [10]. Although there has been gradually increased evidence of IL-27 being an immunosuppressive cytokine in various experimental infection and autoimmune disease models, it is unclear as to the exact role of IL-27 and its effect on retinal autoreactive T cell responses in EAU.

In this study, we immunized IL-27Rα^−/−^ mice and their WT littermates for EAU and compared their disease progression as determined by ocular inflammation and visual function. We also explored their Th1 and Th17 responses by determining the cytokine profile of uveitogenic T cells. We demonstrated that IL-27 signaling deficiency elevated uveitogenic Th1 and Th17 responses as well as their GM-CSF production in EAU mice, associated with exacerbation of ocular inflammation and reduction of visual function. Furthermore, IL-10-producing Tr-1 cell induction was impaired in IL-27Rα^−/−^ EAU mice. Our finding revealed the anti-inflammatory role of IL-27/IL-27R signaling in EAU by regulating multiple T cell lineages and may be extended to treating human uveitis.

## 2. Materials and Methods

### 2.1. Mice

IL-27Rα^−/−^ mice in C57/BL6 background were purchased from Jackson Laboratory (Bar Harbor, ME, USA). IL-27Rα^−/−^ (KO) and their wild-type (WT) littermates were used in the experiments from heterozygote propagation of IL-27Rα^+/−^ mice. All mice were housed in a pathogen-free facility. Animal care and uses in obedience with institutional and ARVO guidelines. Animal experiment protocols were approved and performed under the Animal Care and Use Committee of the Zhongshan Ophthalmic Center.

### 2.2. Induction of EAU

EAU was induced as described previously [29]. IL-27Rα^−/−^ and WT littermates were immunized subcutaneously with 200 μL emulsion of 200 μg human IRBP_1–20_ (GPTHLFQPSLVLDMAKVLLD, Hanhong, China) in an equal volume of complete Freund’s adjuvant (CFA, Sigma-Aldrich, St. Louis, MO, USA) containing 2.5 mg/mL Mycobacterium tuberculosis strain 37RA (Sigma-Aldrich, St. Louis, MO, USA). In addition, these mice also received 0.5 μg of *Bordetella pertussis* toxin (PTX, Sigma-Aldrich, St. Louis, MO, USA) intraperitoneally on the day of immunization and the next day of immunization. 

### 2.3. Clinical Scoring of EAU

The fundus was imaged using the Micron-IV small animal retina imaging system (Phoenix Research Laboratories), as described previously [30]. Clinical EAU severity was scored on a scale of 0–4 on the basis of the number, type, and size of lesions and the extent of inflammation: 0.5—very small peripheral focal lesion, minimal vasculitis/vitritis; 1—mild vasculitis, <5 small focal lesions, ≤1 lineal lesion; 2—multiple chorioretinal lesions and/or infiltrations, severe vasculitis (large size, thick wall, infiltrations), few linear lesions (<5); 3—pattern of linear lesions, large confluent lesions, subretinal neovascularization, retinal hemorrhages, papilledema; 4—large retinal detachment, retinal atrophy [31]. Before acquiring the image, pupils were dilated and local anesthetized using Alcaine drops, tropicamide, and phenylephrine hydrochloride eye drops followed by intraperitoneal injection of a mixture of ketamine and xylazine [30].

### 2.4. Electroretinography (ERG)

Dark-adapted ERG was recorded using Roland consult electrophysiological diagnostic systems (Roland, Germany), as described previously [29,30]. Mice were adapted in the dark for at least 12 h, and the following procedures were performed under dim red light. For the ERG recordings, mice were anesthetized systemically by intraperitoneal injection of a mixture of ketamine and xylazine. Local anesthesia of the pupils was performed using Alcaine drops, tropicamide, and phenylephrine hydrochloride. Electrodes were placed on the center of cornea. Reference and ground electrodes were attached subcutaneously to ears-back area and the posterior neck-back region, respectively. The b-wave amplitude was measured from the trough of the a-wave to the peak of the b-wave.

### 2.5. Isolation of Eye-Infiltrating Cells

Eyes were collected at the peak of disease after active immunization and prepared for flow cytometry analysis separately [32]. After taking the eyes out slowly without orbital tissue, the external conjunctiva and nerve tissue were carefully peeled off. Then, the eyes were dissected along the limbus for lens removal. The remaining tissue was minced by ophthalmic scissors and digested in 1 mg/mL collagenase D (Sigma-Aldrich, St. Louis, MO, USA) with complete culture medium for 50 min at 37 °C. After incubation, the samples were washed, filtered, and re-suspended in cRPMI for following surface and intracellular staining.

### 2.6. Flow Cytometry

Draining lymph nodes (dLNs) and spleen were triturated and dispersed through a 40 μm strainer and then washed with PBS. After washing, single cell suspension was prepared for flow cytometry analysis [6,33]. For surface staining, cells were stained by using anti-mouse CD3 (145-2C11, BD Bioscience, San Jose, CA, USA), CD4 (GK1.5, BD Bioscience), CD8 (53-6.7, BD Bioscience), CD11b (M1/70, BD Bioscience), CD11c (N418, BD Bioscience), and Gr-1 (RB6-BC5, BD Bioscience). For intracellular cytokine staining, cells were incubated for 4 hours with PMA (200 ng/mL, Merck, NJ, USA) and ionomycin (500 mg/mL, Merck) in the presence of Golgistop (BD Bioscience). After stimulation, cells were surface stained as described above. Then, cells were re-suspended with 250 μL fixation solution (BD Bioscience) and washed by permeabilization buffer (BD Bioscience). Twenty minutes later, cells were stained with intracellular antibodies, i.e., anti-IFN-γ (XMG1.2, eBioscience, San Diego, CA, USA), anti-IL-17A (TC11-18H10, BD Bioscience), anti-GM-CSF (MPI-22E9, Biolegend, San Diego, CA, USA), and anti-IL-10 (JES5-16E3, Biolegend) antibodies. For Ki-67 (SoIA15, eBioscience) staining, cells were fixed and permeabilized by transcription factor staining buffer set (eBioscience) for nuclear protein staining. Results were analyzed by FlowJo (Tree star Inc., Oakland, CA, USA).

### 2.7. T Cell Activation and Differentiation

The dLNs were harvested from WT and KO mice at peak of EAU disease. Single cell suspensions were prepared as described above. Cells were counted and cultured with or without IRBP_1–20_ peptide at a density of 2.5 × 10^6^ cells/mL in 96-well U-bottom plates for 48h at 37 °C. Supernatants was collected for cytokine determination using ELISA (Invitrogen, San Diego, CA, USA) according to the manufacturer’s instructions.

CD4^+^CD62L^+^ T cells were sorted from spleen cells by cell sorter (FACSAriaIII Fusion, BD Bioscience). They were stimulated by plate bound anti-CD3 (2 μg/mL) and soluble anti-CD28 (1 μg/mL) antibodies. For Th17 polarization, culture media were supplemented with IL-6 (25 ng/mL), TGF-β (2.5 ng/mL), anti-IFN-γ (10 μg/mL), and anti-IL-4 (10 μg/mL) antibodies. On day 3, cells were pulsed with PMA and ionomycin in the presence of Golgistop for 4 h. The cytokine expression was measured as described by flow cytometry.

### 2.8. Real-Time PCR

CD4^+^ T cells (purity >95%) were isolated from spleen using cell sorter (FACSAria Fusion, BD Bioscience). Total RNA extraction was carried out with RNAiso Plus reagent (Takara, Tokyo, Japan) according to the manufacturer’s instructions. RNA quality and concentration were determined by NanoDrop 2000 (Thermo Fisher Scientific, Waltham, MA, USA), and cDNA was synthesized with FastKing-RT SuperMix kit (Tiangen, Beijing, China). Quantitative real-time PCR was performed with ABI step-one plus system using gene-specific primer from Thermo Fisher (Thermo Fisher Scientific). Primers used for quantitative PCR are listed in Table 1. All data were normalized to glyceraldehyde 3 phosphate dehydrogenase (GAPDH) expression and expressed relative to WT CD4^+^ T cells. 

### 2.9. Statistical Analysis

All data were expressed as means ± SEM. Unpaired *t*-test and two-way ANOVA analysis were used for two groups and multiple groups comparison, respectively (Graphpad Prism 6, GraphPad Software, Inc., La Jolla, CA, USA). The statistical significance was considered when *p* < 0.05.

## 3. Results

### 3.1. IL-27Rα^−/−^ Mice Developed More Severe EAU Than WT Littermates

**Figure 1:**
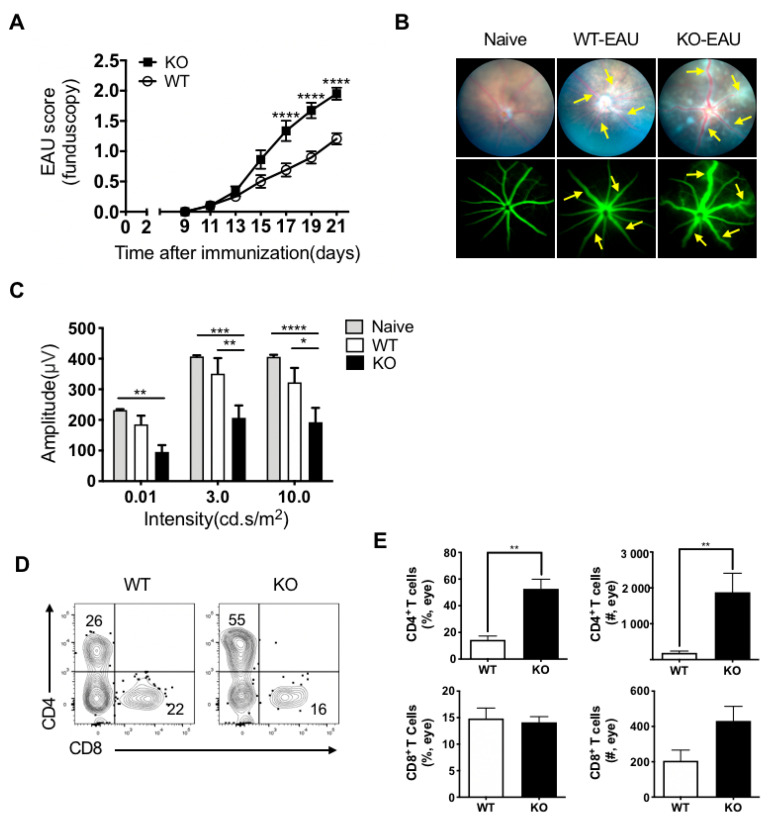
IL-27Ra^−/−^ mice developed more severe EAU than their WT littermates. EAU was induced in IL-27Rα^−/−^ (KO) mice and their WT littermates by active immunization with retinal antigen IRBP_1–20_ peptide and CFA in the presence of PTx. (**A**) Clinical score of EAU in KO mice and WT littermates. Data represent the means ± SEMs (WT: n = 22 eyes; KO: n = 20 eyes). **** *p* < 0.0001, two-way ANOVA. (**B**) Representative retinal images assessed by funduscopy (upper) and fluorescein angiography (bottom) from naïve, WT, and KO EAU mice at the peak of disease on day 21 post-immunization. Vasculitis and perivascular cellular exudates are marked by arrows. (**C**) The b-wave amplitude of scotopic ERG response among naïve, WT, and KO mice at the peak of EAU (day 21). Data represent the means ± SEMs from a representative of three independent experiments, and each group contained at least 6 samples. * *p* < 0.05, ** *p* < 0.01, *** *p* < 0.001, **** *p* < 0.0001, two-way ANOVA. (**D**,**E**) Inflammatory cells were isolated from EAU eyes at the peak of disease. (**D**) Representative FCM plots. (**E**) Frequency (%) and actual number (#) of CD4^+^ and CD8^+^ T cells were assessed in the eyes from WT and KO EAU mice. Data represent the means ± SEMs (WT: n = 5 eyes; KO: n = 9 eyes). ** *p* < 0.01, Mann–Whitney *U* test.

To investigate the role of IL27/IL-27R signaling in EAU, we immunized IL-27Rα^−/−^ and their WT littermates by subcutaneous injection of retinal antigen IRBP_1–20_ peptide emulsified in CFA with co-administration of PTx. We evaluated the kinetics of disease development by fundus examination with the Micron-IV small animal retina imaging system (Figure 1A). EAU onset was on days 11–14 post-immunization. We observed that EAU scores of IL-27Rα^−/−^ mice were significantly higher than their WT littermates on days 17, 19, and 21 (*p* < 0.0001, Figure 1A). Retinal inflammatory activity was monitored by funduscopy and fluorescein angiography at different time points. We found that the eyes of IL-27Rα^−/−^ mice exhibited more severe vasculitis/retinitis and perivascular exudates by day 21 at the peak of EAU, WT littermates developed focal lesions around optic nerve and less inflammatory exudates at the same time, and naïve WT mice served as the healthy control (Figure 1B).

We further evaluated the visual function of EAU immunized IL-27Rα^−/−^ mice and their WT littermates by electroretinogram (ERG) with different stimulation intensities after overnight dark adaptation at day 21 post-immunization (Figure 1C). We found that the IL-27Rα^−/−^ mice showed significantly lower ERG amplitudes than their WT littermates (Figure 1C). Consistently, we found that CD4^+^ T cell (but not CD8^+^ T cell) infiltration dramatically increased in the eyes of IL-27Rα^−/−^ EAU mice, both in frequency and actual number (Figure 1D,E). In addition, we observed the increase of myeloid cell infiltration in IL-27Rα^−/−^ EAU mice (Figure S1). The proliferation of eye-infiltrating CD4^+^ T cells and myeloid cells was determined by Ki-67, and no statistically significant difference was observed (Figure S2). These observations concluded that deficiency of IL27/IL-27R signaling exacerbated EAU, as illustrated by increased ocular inflammation, reduced visual function, and increased immune cell infiltration.

### 3.2. Uveitogenic Th1 and Th17 Responses Were Elevated in IL-27Rα^−/−^ EAU Mice

**Figure 2:**
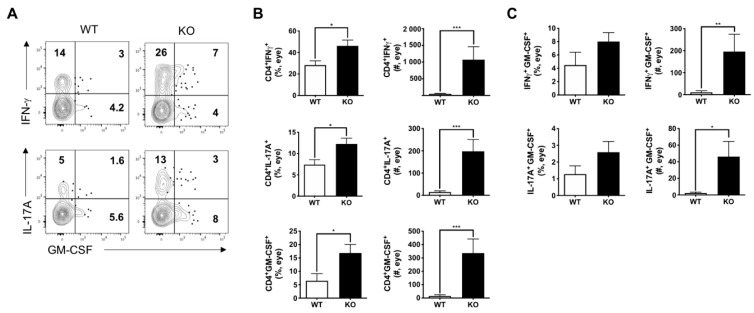
Ocular Th1 and Th17 responses increased in IL-27Rα^−/−^ EAU mice. Inflammatory cells were isolated from the eyes from WT and KO EAU mice at the peak of disease and assessed for FCM analysis. (**A**) Representative plots of IFN-γ, IL-17A, and GM-CSF production in the inflamed eyes. Cells were gated on CD4^+^ population. (**B**) Frequency (%) and actual number (#) of IFN-γ-, IL-17A-, and GM-CSF-producing CD4^+^ T cells in the EAU eyes. (**C**) Frequency (%) and actual number (#) of IFN-γ^+^ GM-CSF^+^ and IL-17A^+^GM-CSF^+^ dbl-pos CD4^+^ T cells in the EAU eyes. Data represent the means ± SEMs (WT: n = 5 eyes; KO: n = 9 eyes), * *p* < 0.05, ** *p* < 0.01, *** *p* < 0.001, Mann–Whitney *U* test.

Next, we compared the expression level of Th1 and Th17 signature cytokines, i.e., IFN-γ and IL-17A, in eye-infiltrating CD4^+^ T cells from EAU-immunized IL-27Rα^−/−^ mice and their WT littermates. Consistent with the increased ocular inflammation (Figure 1), we found that the infiltration of IL-17A- and IFN-γ-producing CD4^+^ T cells were significantly increased in the eyes of IL-27Rα^−/−^ EAU mice (Figure 2A,B).

Notably, GM-CSF was identified as another critical pathogenic cytokine in CNS autoimmune diseases because of its strong pathophysiological consequences shared by both Th1 and Th17 response, and recent studies have suggested that it can be suppressed by IL-27 [27,34,35,36]. We therefore investigated its production by eye-infiltrating CD4^+^ T cells and confirmed that there was an increase of GM-CSF-producing CD4^+^ T cells in IL-27Rα^−/−^ EAU mice (Figure 2A,B). Interestingly, this elevation of GM-CSF was found in both Th1 and Th17 cells, as both number of IFN-γ^+^GM-CSF^+^ and IL-17A^+^GM-CSF^+^ CD4^+^ T cells were increased (Figure 2A,C). The appearance of these inflammatory cytokine double-positive CD4^+^ T cells suggests that IL-27 signaling deficiency leads to increase of pathogenicity of eye-infiltrating Th1 and Th17 cells, which aggravates EAU disease.

### 3.3. Peripheral Th1 and Th17 Responses Increased in IL-27Rα^−/−^ EAU Mice

**Figure 3:**
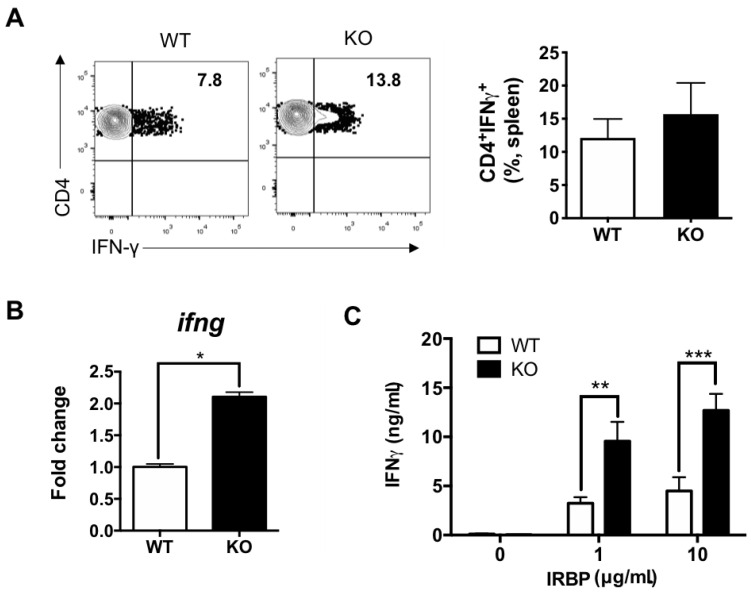
Peripheral Th1 response increased in IL-27Ra^−/−^ EAU mice. (**A**) Representative plots and frequency of IFN-γ-producing CD4^+^ T cells in the spleens from WT and KO EAU mice. Data represent the means ± SEMs of two experiments (WT: n = 5 mice; KO: n = 7 mice). (**B**) The relative gene expression of *ifng* in CD4^+^ T cells. Total RNA was extracted from CD4^+^ T cells that were sorted from the spleens of WT and KO EAU mice on day 21 post-immunization. Data represent the means ± SEMs from a representative of two independent experiments (4 mice per group). * *p* < 0.05, Mann–Whitney *U* test. (**C**) Retinal antigen-specific IFN-γ production in dLNs of WT and KO EAU mice. Lymphocytes were isolated from dLNs and stimulated with IRBP for 48 h. Cultured supernatants were collected for cytokine determination by ELISA. Data represent the means ± SEMs of two experiments (WT: n = 5 mice; KO: n = 6 mice). ** *p* < 0.01, *** *p* < 0.001, two-way ANOVA.

**Figure 4:**
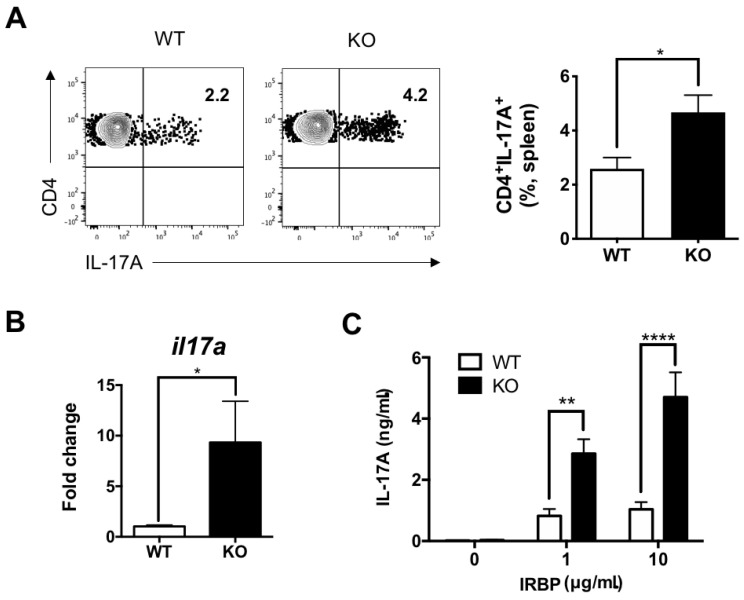
Peripheral Th17 response increased in IL-27Ra^−/−^ EAU mice. (**A**) Representative plots and frequency of IL-17A-producing CD4^+^ T cells in the spleens from WT and KO EAU mice. Data represent the means ± SEMs of two experiments (WT: n = 5 mice; KO: n = 7 mice). * *p* < 0.05, Mann–Whitney *U* test. (**B**) The relative gene expression of *il17a* in CD4^+^ T cells sorted from the spleens of WT and KO EAU mice. Total RNA was extracted from CD4^+^ T cells that were sorted from the spleens of mice on day 21 post-immunization. Data represent the means ± SEMs from a representative of two experiments (4 mice per group). * *p* < 0.05, Mann–Whitney *U* test. (**C**) Retinal antigen-specific IL-17A production in dLNs of WT and KO EAU mice. Lymphocytes were isolated from dLNs and stimulated with IRBP for 48 h. Cultured supernatants were collected for cytokine determination by ELISA. Data represent the means ± SEMs of two experiments (WT: n = 5 mice; KO: n = 6 mice). ** *p* < 0.01, **** *p* < 0.0001, two-way ANOVA.

To further confirm the elevation of autoreactive Th1 and Th17 responses in IL-27Rα^−/−^ EAU mice, we investigated the peripheral Th1 and Th17 responses by studying the IFN-γ and IL-17A production by CD4^+^ T cells from the spleen, which is the largest secondary lymphoid organ. We first determined the total CD4^+^ T cells response by PMA/ionomycin stimulation, which was then followed by intracellular cytokine staining. We observed that there is a trend of increase of IFN-γ-producing CD4^+^ T cells in the spleen of IL-27Rα^−/−^ EAU mice when compared to their WT littermates, although it did not reach statistical significance (Figure 3A). However, the number of IL-17A-producing CD4^+^ T cells was significantly increased in IL-27Rα^−/−^ EAU mice (Figure 4A). Consistent with previous studies [37], there is a trend of lower IFN-γ and higher IL-17A production by splenic CD4^+^ T cells in naïve IL-27Rα^−/−^ mice when compared to their WT littermates (Figure S3).

We next studied the mRNA expression of *ifng* and *il17a* in sorted splenic CD4^+^ T cells, and the expression level was significantly higher in IL-27Rα^−/−^ EAU mice when compared to their WT littermates (Figure 3B and Figure 4B). Then, we investigated the IRBP-specific Th1 and Th17 responses from the draining lymph nodes (dLNs) because these are the site for T cell priming before they migrate to the inflamed tissue for disease development. We stimulated the lymphocytes isolated from dLNs of EAU mice with IRBP_1–20_ peptide and determined the production of IL-17A and IFN-γ by ELISA. We found that lymphocytes obtained from IL-27Rα^−/−^ EAU mice produced significantly higher IFN-γ and IL-17A than their WT littermates (Figure 3C and Figure 4C). These data concluded that IL-27 signaling exerted a suppressive effect on the peripheral uveitogenic Th1 and Th17 responses.

### 3.4. IL-27 Suppressed GM-CSF Production from Effector T Cells

**Figure 5:**
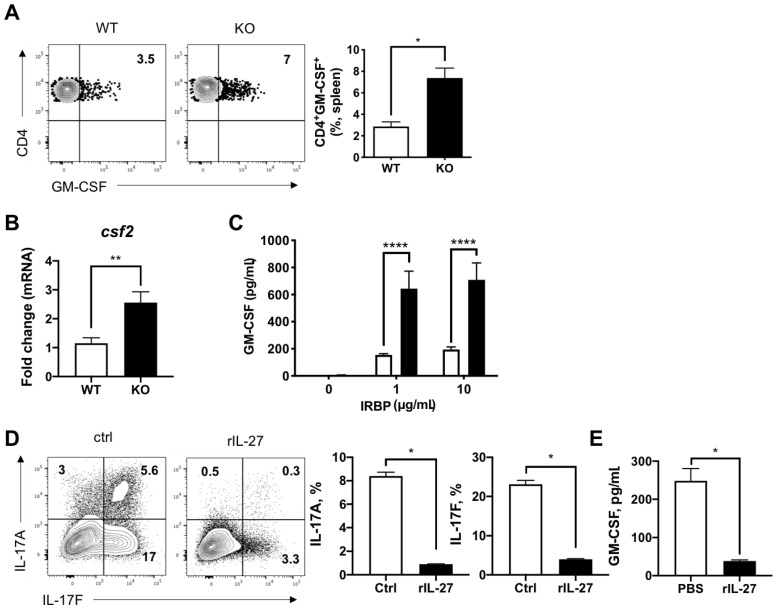
Production of GM-CSF increased in IL-27Ra^−/−^ EAU mice. (**A**) Representative plots and frequency of GM-CSF-producing CD4^+^ T cells in the spleens from WT and KO EAU mice. Data represent the means ± SEMs of two experiments (WT: n = 5 mice; KO: n = 7 mice). * *p* < 0.05, Mann–Whitney *U* test. (**B**) The relative gene expression of *csf2* in CD4^+^ T cells sorted from the spleens of WT and KO EAU mice. Total RNA was extracted from CD4^+^ T cells that were sorted from the spleens of EAU mice on day 21 post-immunization. Data represent the means ± SEMs from a representative of two experiments (4 mice per group). ** *p* < 0.01, Mann–Whitney *U* test. (**C**) Retinal antigen-specific GM-CSF production in dLNs of WT and KO EAU mice. Lymphocytes were isolated from dLNs and stimulated with IRBP for 48 h. Cultured supernatants were collected for cytokine determination by ELISA. Data represent the means ± SEMs of two experiments (WT: n = 5 mice; KO: n = 6 mice). **** *p* < 0.0001, two-way ANOVA. (**D**,**E**) CD4^+^CD62L^+^ naïve T cells were purified and polarized under Th17 condition with aCD3/CD28 antibodies in the presence of recombinant IL-27 protein for 3 days. For Th17 polarization, culture media were supplemented with IL-6 (25 ng/mL), TGF-β (2.5 ng/mL), anti-IFN-γ (10 μg/mL), and anti-IL-4 (10 μg/mL) antibodies. (**D**) Representative FCM plots and frequency of IL-17A and IL-17F expression in CD4 T cells are shown. (**E**) GM-CSF in the cultured supernatants was determined by ELISA at the same time. Data represent the means ± SEMs of three combined experiments. * *p* < 0.05, two-way ANOVA.

GM-CSF has been shown to be an important pathogenic cytokine from Th17 cells in neuroinflammation [27]. Therefore, we also examined the GM-CSF protein and mRNA expression from splenic CD4^+^ T cells in EAU mice. Both findings showed that GM-CSF production was significantly increased in IL-27Rα^−/−^ EAU mice when compared to their WT littermates (Figure 5A,B). Consistently, the IRBP-specific GM-CSF production from the cells obtained from the dLNs of IL-27Rα^−/−^ EAU mice was also elevated (Figure 5C).

To further confirm the role of IL-27 to suppress GM-CSF production from autoreactive CD4^+^ T cells, we polarized naïve CD4^+^ T cells into Th17 cells under type-specific condition with or without purified recombinant IL-27 and observed that IL-27 significantly suppressed their production of GM-CSF (Figure 5D). These data suggested that IL-27 effectively inhibits GM-CSF production from autoreactive CD4^+^ T cells, which may explain the exacerbated EAU in IL-27Rα^−/−^ mice.

### 3.5. IL-27Rα Deficiency Impaired Tr1 Cell Induction

**Figure 6:**
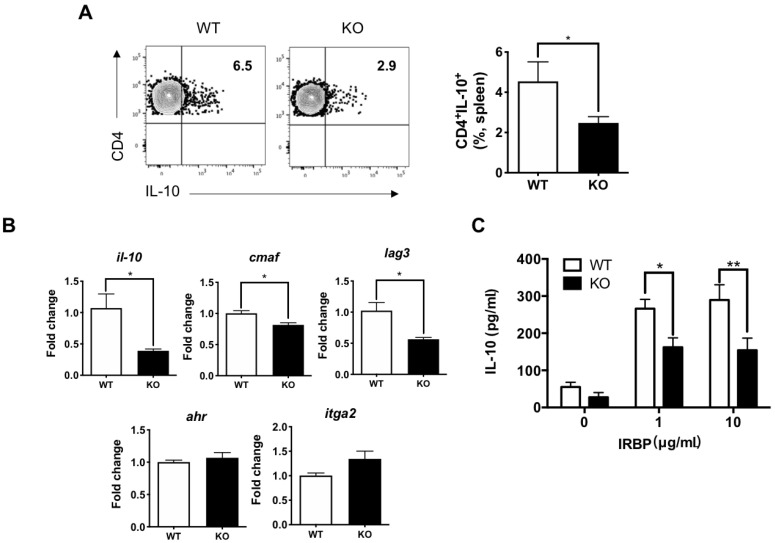
Deficiency of IL-27Rαa impaired Tr1 cell induction. (**A**) Representative plots and frequency of IL-10-producing CD4^+^ T cells in the spleens from WT and KO EAU mice. Data represent the means ± SEMs of two experiments (WT: n = 5 mice; KO: n = 7 mice). * *p* < 0.05, Mann–Whitney *U* test. (**B**) The relative gene expression of *il-10*, *cmaf*, *lag3*, *ahr*, and *itga2* in CD4^+^ T cells sorted from the spleens of WT and KO EAU mice. Total RNA was extracted from CD4^+^ T cells that were sorted from the spleens of EAU mice on day 21 post-immunization. Data represent the means ± SEMs from a representative of two independent experiments (4 mice per group). * *p* < 0.05, Mann–Whitney *U* test. (**C**) Retinal antigen-specific IL-10 production in dLNs of WT and KO EAU mice. Lymphocytes from dLNs of EAU mice were isolated and stimulated with IRBP for 48 h. Cultured supernatants were collected for cytokine determination by ELISA. Data represent the means ± SEMs of three experiments (WT: n = 9 mice; KO: n = 10 mice). * *p* < 0.05, ** *p* < 0.01, two-way ANOVA.

IL-27 is an important cytokine to induce Tr1 cells [38,39], which are characterized by production of IL-10. Tr1 cells have been identified to play a crucial role in maintaining immune tolerance and suppressing tissue inflammation [35,40]. We next examined the effect of IL-27 in regulating Tr1 cell induction in IL-27Rα^−/−^ mice after EAU immunization, by assessment of IL-10 expression on CD4^+^ T cells. We found that IL-10-producing CD4^+^ T cells were significantly decreased in the spleen of IL-27Rα^−/−^ EAU mice in comparison to those from their WT littermates (Figure 6A). In addition, the IRBP-specific IL-10 production was significantly decreased in IL-27Rα^−/−^ EAU mice compared to those in WT controls (Figure 6B). 

To further confirm the impaired induction of IL-10-producing Tr1 cells in IL-27Rα^−/−^ mice, we sorted CD4^+^ T cells from the spleen of IL-27Rα^−/−^ mice and their WT littermates at day 21 after EAU immunization and investigated their expression of Tr1-related genes, including *il10*, *cmaf*, *lag3*, *ahr*, and *itga2* [41,42,43]. We found that the expression of *il10*, *cmaf*, and *lag3*, but not *ahr* and *itga2*, was significantly decreased in CD4^+^ T cells from IL-27Rα^−/−^ mice with EAU in comparison to WT littermates (Figure 6C). We therefore concluded that IRBP-specific IL-10 production and Tr1 cell induction were both impaired in IL-27Rα^−/−^ mice and could account for the exacerbation of EAU.

It is of note that a natural induction of regulatory T cell (Treg) brings about resolution of EAU [44]. Taken into consideration that IL-27 could also regulate Treg [45], we determined the Treg master transcriptional factor, Foxp3, in splenic CD4^+^ T cells, and no difference were observed between IL-27Rα^−/−^ mice and their WT littermates (Figure S4). This data suggested that IL-27 signaling does not alter Treg cells in EAU.

## 4. Discussion

In this study, we reported that IL-27/IL-27R signaling had an anti-inflammatory role in EAU. This signaling delineated the differences between IL-27Rα^−/−^ mice and their WT littermates with EAU from multiple aspects, including disease severity, visual electrophysiology, and cellular and molecular immunology. Exacerbated disease exhibited severer ocular inflammation, and vision loss was observed in IL-27Rα^−/−^ mice. The protective function of IL-27 has been studied in EAE [46,47,48]. However, EAU has its own features on pathogenesis, which is different from other CNS experimental autoimmune diseases models [49]. Therefore, it is necessary to investigate the unique function of IL-27/IL-27R signaling in EAU. In this study, we revealed that this signal is required to regulate uveitogenic Th1 and Th17 responses and promote the induction of the suppressive Tr1 cells in EAU. These results provided further evidence to support the therapeutic potential of IL-27 for better control of uveitis, as well as other autoimmune diseases.

The role of IL-27 signaling in regulating Th1 response is relatively controversial. The effect of IL-27 to promote early Th1 response has been well established because of its capacity to induce IL12Rβ2 [50]. However, later studies confirmed that IL-27Rα^−/−^ mice developed normal Th1 response after infection [51], and IL-27 could induce IL-10 and SOCS3 to suppress Th1 cells, suggesting the bidirectional role of IL-27 in Th1 cells [51,52,53]. These works are consistent with our observation that IL-27Rα^−/−^ EAU mice developed elevated Th1 response.

There is growing evidence to support that autoreactive Th17 cells and their Th17 lineage cytokine are the dominant contributors for the development of autoimmune diseases, including uveitis [11,54,55]. Early studies reported that IL-27 suppresses the expression of RORα and RORγ and inhibits the differentiation of Th17 cells [56]. Further studies confirmed that IL-27 inhibits the production of IL-17A from Th17 cells through STAT1 [46,47,55]. In this study, we found that deficient of IL-27 signaling leads to the increase of IL-17A-producing ocular infiltrating CD4^+^ cells, as well as the retinal antigen-specific Th17 response in mice with EAU. Therefore, IL-27 is an essential negative regulator for Th17 response in EAU pathogenesis.

Taken together, IL-27 signaling is crucial in dampening the pathogenic Th1 and Th17 response during EAU development, and absence of the cytokine leads to worsen ocular inflammation and visual impairment. Different from us and others, Sonoda et al. [57] reported that EAU immunized IL-27Rα^−/−^ mice displayed delay onset when compared to WT mice because of the impaired Th1 response, having yet to involve other Th lineage observations. This discrepancy could be explained by the microbiota differences between the IL-27Rα^−/−^ and WT mice that they studied. Microbiome has been shown to contribute to the disease susceptibility of the host. In order to minimize the contribution of the difference in microbiome to the susceptibility of the IL-27Rα^−/−^ and WT mice to EAU, we therefore used WT littermates as the control group in EAU experiments and confirmed that IL-27 signaling plays a protective role in EAU. In addition, in vivo delivery of recombinant IL-27 by different approaches, including protein infusion pump, as well as lentiviral and adeno-associated virus packaging, have been used to confirm the therapeutic effect of IL-27 in suppressing experimental autoimmune diseases [46,49,58,59].

GM-CSF has been identified as a pathogenic cytokine in autoimmune including colitis [34], arthritis [60], and multiple sclerosis [61]. It is one of few cytokines whose deletion individually could protect from EAE development and monoclonal antibody against GM-CSF have entered into clinical trial [62]. Some researchers even considered GM-CSF-producing T cells as a new Th subset that distinct from the Th1 or Th17 [63]. In EAU, GM-CSF-producing CD4^+^ T cells is enough to drive ocular inflammation at the absence of IFN-γ and IL-17A by driving eosinophil infiltration [64]. GM-CSF is also critical for the pathogenicity of Th1 and Th17 cells in EAE [36]. Previous studies reported that IL-27 acted as the negative regulator of GM-CSF through Jak2/Tyk2 and STAT1 signaling [27,48,65]. Consistently, we also observed that, similar to IFN-γ and IL-17A, GM-CSF was highly expressed by the eye-infiltrating CD4^+^ T cells of IL-27Rα^−/−^ mice when compared to their WT littermates. In addition, we confirmed the robust suppressive effect of IL-27 on GM-CSF production from Th17 polarized CD4^+^ T cells in vitro. 

IL-10 is a suppressive cytokine that protects mice from EAU. IL-10^−/−^ mice were found to be more susceptible to EAU [66], while overexpression of IL-10 ameliorated disease progression [67]. More importantly, our previous study demonstrated that WT Tr1 cells suppressed EAU better than IL27Rα^−/−^ Tr1 cells after being adoptively transferred to the animals [14]. Previous studies confirmed the important role of IL-27 in the induction of IL-10-producing Tr1 cells to resolve autoimmune diseases, especially in EAE [46,68]. IL-27 induces Tr1 cells by promoting the phosphorylation of STAT1 and STAT3 [69], which induce the expression of c-maf, IL-21, and inducible costimulator (ICOS) [70]. A later study further identified the role of c-maf in this event by demonstrating its ability to couple with Ahr for optimal production of IL-10 [44]. In line with these studies, our data showed that deficient in IL-27 signaling impairs the antigen-specific IL-10 response and IL-10-producing Tr1 cell induction with downregulation of Tr1-related genes, including *il10*, *cmaf*, and *lag3* in splenic CD4^+^ T cells from IL-27Rα^−/−^ EAU mice. Although other Tr1-related genes, *ahr* and *itga2*, displayed similar expression in IL-27Rα^−/−^ mice and their WT littermates, they were also expressed by Th17 cells, Treg cells, and NKT cells [68,71]. We also showed that IL-10-producing CD4^+^ T cells were significantly decreased in the spleen of IL-27Rα^−/−^ EAU mice. Our previous study already identified the important role of the NK-DC interaction in regulating autoreactive Th17 response in EAU by promoting Tr1 cells through IL-27, and IL-27Rα^−/−^ T cells are less capable of being differentiated into Tr1 cells [14]. We suggested that IL-27-induced IL-10^+^ Tr1 cells are critical to inhibit the priming of Th17 response during EAU development. It should be noted that these regulatory properties of IL-27 are extendable to the human system. IL-27 has been demonstrated to suppress human Th17 cells and promote human Tr1 cells, suggesting the important role of IL-27 in regulating human autoimmune diseases [72,73].

Interestingly, there is a gender difference present, with a higher susceptibility of non-infectious uveitis, as well as other autoimmune diseases, in women [74]. Many studies focus the role of sex hormones in this aspect, while recent investigations have revealed a potential role of different regulation in immune gene expression in women that may explain this phenomenon, not only in autoimmune diseases, but also in infectious diseases. Future studies will be required for better clarification [75].

Uveitis is a complex disease that involves dysregulation of multiple T cell subsets and their signature cytokines [76]. The elevation of these proinflammatory cytokines, including IFN-γ, IL-17, and GM-CSF, has been reported in patients with uveitis [76,77]. Our data support the potential role of IL-27 in treating autoimmune uveitis by countering these proinflammatory cytokines. In addition, we showed that IL-27 signaling is important for the induction of IL-10-producing Tr1 cell, which is also impaired in patients with autoimmune diseases, including MS [36], suggesting that IL-27 could help to restore immune tolerance in patients with autoimmune diseases by promoting this regulatory T cell subset.

In conclusion, we demonstrated that IL-27 signaling deficiency leads to increase of Th1 and Th17 response and decrease of Tr1 induction for the pathogenesis of EAU. Notably, IL-27 suppresses the production of pathogenic GM-CSF from Th cells both in vivo and in vitro. This provides new insight for potential therapy for uveitis by targeting IL-27 signaling.

## Figures and Tables

**Table 1:** Forward and reverse primers. Primers are shown for each gene analyzed by real-time PCR.

Mouse Gene		Primer (5′ to 3′)
*il17a*	Forward	TTTAACTCCCTTGGCGCAAAA
	Reverse	CTTTCCCTCCGCATTGACAC
*ifng*	Forward	TCAAGT GGCATAGATGTGGAAGAA
	Reverse	TGGCTCTGCAGGATTTTCATG
*il10*	Forward	AACTGCACCCACTTCCCAGTC
	Reverse	CATTAAGGAGTCGGTTAGCAG
*cmaf*	Forward	GCAACGGCTTCCGAGAAAAC
	Reverse	CCCCCACGGAGCATTTAACA
*lag3*	Forward	TCTCATCCTTGGTGCCCTCT
	Reverse	CCGGAAATGGCTGAATCCCA
*ahr*	Forward	AGCCGGTGCAGAAAACAGTA
	Reverse	CCAGGCGGTCTAACTCTGTG
*itga2*	Forward	TCAGGTGGGCACAACTCTTC
	Reverse	GAGGGGCAAACGGTAGGAAA
*foxp3*	Forward	CCCATCCCCAGGAGTCTTG
	Reverse	ACCATGACTAGGGGCACTGTA

## Data Availability

The data analyzed during the current study are available from the corresponding author on reasonable request.

## Supplementary Materials

The following are available online at https://www.mdpi.com/article/10.3390/ijms22147517/s1.

## Abbreviations

APCs	antigen-presenting cells
CFA	complete Freund’s adjuvant
CNS	central nervous system
dLNs	draining lymph nodes
EAE	experimental autoimmune encephalomyelitis
EAU	experimental autoimmune uveitis
GAPDH	glyceraldehyde 3 phosphate dehydrogenase
GM-CSF	granulocyte-macrophage colony-stimulating factor
PTx	Bordetella pertussis toxin
Tr1 cells	T regulatory type 1 cells

## References

[1] Horai R., Caspi R.R. (2019). Microbiome and Autoimmune Uveitis. Front. Immunol..

[2] Nussenblatt R.B. (1990). The natural history of uveitis. Int. Ophthalmol..

[3] Caspi R.R., Roberge F.G., Chan C.C., Wiggert B., Chader G.J., Rozenszajn L.A., Lando Z., Nussenblatt R.B. (1988). A new model of autoimmune disease. Experimental autoimmune uveoretinitis induced in mice with two different retinal antigens. J. Immunol..

[4] Caspi R.R., Chan C.C., Wiggert B., Chader G.J. (1990). The mouse as a model of experimental autoimmune uveoretinitis (EAU). Curr. Eye Res..

[5] Chan C.C., Caspi R.R., Ni M., Leake W.C., Wiggert B., Chader G.J., Nussenblatt R.B. (1990). Pathology of experimental autoimmune uveoretinitis in mice. J. Autoimmun..

[6] Horai R., Silver P.B., Chen J., Agarwal R.K., Chong W.P., Jittayasothorn Y., Mattapallil M.J., Nguyen S., Natarajan K., Villasmil R. (2013). Breakdown of immune privilege and spontaneous autoimmunity in mice expressing a transgenic T cell receptor specific for a retinal autoantigen. J. Autoimmun..

[7] Oh H.M., Yu C.R., Lee Y., Chan C.C., Maminishkis A., Egwuagu C.E. (2011). Autoreactive memory CD4+ T lymphocytes that mediate chronic uveitis reside in the bone marrow through STAT3-dependent mechanisms. J. Immunol..

[8] Steinman L. (2007). A brief history of T(H)17, the first major revision in the T(H)1/T(H)2 hypothesis of T cell-mediated tissue damage. Nat. Med..

[9] Luger D., Silver P.B., Tang J., Cua D., Chen Z., Iwakura Y., Bowman E.P., Sgambellone N.P., Chan C.-C., Caspi R.R. (2008). Either a Th17 or a Th1 effector response can drive autoimmunity: Conditions of disease induction affect dominant effector category. J. Exp. Med..

[10] Wang C., Tian Y., Lei B., Xiao X., Ye Z., Li F., Kijlstra A., Yang P. (2012). Decreased IL-27 expression in association with an increased Th17 response in Vogt-Koyanagi-Harada disease. Investig. Ophthalmol. Vis. Sci..

[11] Chi W., Zhu X., Yang P., Liu X., Lin X., Zhou H., Huang X., Kijlstra A. (2008). Upregulated IL-23 and IL-17 in Behcet patients with active uveitis. Investig. Ophthalmol. Vis. Sci..

[12] Yi T., Zhao D., Lin C.L., Zhang C., Chen Y., Todorov I., LeBon T., Kandeel F., Forman S., Zeng D. (2008). Absence of donor Th17 leads to augmented Th1 differentiation and exacerbated acute graft-versus-host disease. Blood.

[13] Egwuagu C.E., Sztein J., Mahdi R.M., Li W., Chepelinsky A.B. (1999). IFN-γ Increases the Severity and Accelerates the Onset of Experimental Autoimmune Uveitis in Transgenic Rats. J. Immunol..

[14] Chong W.P., van Panhuys N., Chen J., Silver P.B., Jittayasothorn Y., Mattapallil M.J., Germain R.N., Caspi R.R. (2015). NK-DC crosstalk controls the autopathogenic Th17 response through an innate IFN-gamma-IL-27 axis. J. Exp. Med..

[15] Grajewski R.S., Hansen A.M., Agarwal R.K., Kronenberg M., Sidobre S., Su S.B., Silver P.B., Tsuji M., Franck R.W., Lawton A.P. (2008). Activation of invariant NKT cells ameliorates experimental ocular autoimmunity by a mechanism involving innate IFN-gamma production and dampening of the adaptive Th1 and Th17 responses. J. Immunol..

[16] Ferber I.A., Brocke S., Taylor-Edwards C., Ridgway W., Dinisco C., Steinman L., Dalton D., Fathman C.G. (1996). Mice with a disrupted IFN-gamma gene are susceptible to the induction of experimental autoimmune encephalomyelitis (EAE). J. Immunol..

[17] Patel D.D., Kuchroo V.K. (2015). Th17 Cell Pathway in Human Immunity, Lessons from Genetics and Therapeutic Interventions. Immunity.

[18] Chong W.P., Mattapallil M.J., Raychaudhuri K., Bing S.J., Wu S., Zhong Y., Wang W.W., Chen Z., Silver P.B., Jittayasothorn Y. (2020). The Cytokine IL-17A Limits Th17 Pathogenicity via a Negative Feedback Loop Driven by Autocrine Induction of IL-24. Immunity.

[19] Chong W.P., Horai R., Mattapallil M.J., Silver P.B., Chen J., Zhou R., Sergeev Y., Villasmil R., Chan C.-C., Caspi R.R. (2014). IL-27p28 inhibits central nervous system autoimmunity by concurrently antagonizing Th1 and Th17 responses. J. Autoimmun..

[20] Chen Q., Ghilardi N., Wang H., Baker T., Xie M.H., Gurney A., Grewal I.S., de Sauvage F.J. (2000). Development of Th1-type immune responses requires the type I cytokine receptor TCCR. Nature.

[21] Sonobe Y., Yawata I., Kawanokuchi J., Takeuchi H., Mizuno T., Suzumura A. (2005). Production of IL-27 and other IL-12 family cytokines by microglia and their subpopulations. Brain Res..

[22] Pflanz S., Timans J.C., Cheung J., Rosales R., Kanzler H., Gilbert J., Hibbert L., Churakova T., Travis M., Vaisberg E. (2002). IL-27, a heterodimeric cytokine composed of EBI3 and p28 protein, induces proliferation of naive CD4+ T cells. Immunity.

[23] Yoshida H., Hamano S., Senaldi G., Covey T., Faggioni R., Mu S., Xia M., Wakeham A.C., Nishina H., Potter J. (2001). WSX-1 Is Required for the Initiation of Th1 Responses and Resistance to L. major Infection. Immunity.

[24] Yamanaka A., Hamano S., Miyazaki Y., Ishii K., Takeda A., Mak T.W., Himeno K., Yoshimura A., Yoshida H. (2004). Hyperproduction of Proinflammatory Cytokines by WSX-1-Deficient NKT Cells in Concanavalin A-Induced Hepatitis. J. Immunol..

[25] Yang J., Yang M., Htut T.M., Ouyang X., Hanidu A., Li X., Sellati R., Jiang H., Zhang S., Li H. (2008). Epstein-Barr virus-induced gene 3 negatively regulates IL-17, IL-22 and RORγt. Eur. J. Immunol..

[26] El-behi M., Ciric B., Yu S., Zhang G.X., Fitzgerald D.C., Rostami A. (2009). Differential effect of IL-27 on developing versus committed Th17 cells. J. Immunol..

[27] Codarri L., Gyülvészi G., Tosevski V., Hesske L., Fontana A., Magnenat L., Suter T., Becher B. (2011). RORγt drives production of the cytokine GM-CSF in helper T cells, which is essential for the effector phase of autoimmune neuroinflammation. Nat. Immunol..

[28] Jiang S., Liu X., Luo L., Qu B., Huang X., Xu L., Lin Y., Ye S., Liu Y. (2010). Elevated serum IL-23 correlates with intraocular inflammation after cataract surgery in patients with Vogt-Koyanagi-Harada disease. Br. J. Ophthalmol..

[29] Chen J., Qian H., Horai R., Chan C.C., Falick Y., Caspi R.R. (2013). Comparative analysis of induced vs. spontaneous models of autoimmune uveitis targeting the interphotoreceptor retinoid binding protein. PLoS ONE.

[30] Chen Y., Chen Z., Chong W.P., Wu S., Wang W., Zhou H., Chen J. (2018). Comparative Analysis of the Interphotoreceptor Retinoid Binding ProteinInduced Models of Experimental Autoimmune Uveitis in B10.RIII versus C57BL/6 Mice. Curr. Mol. Med..

[31] Agarwal R.K., Silver P.B., Caspi R.R. (2012). Rodent Models of Experimental Autoimmune Uveitis. Methods Mol. Biol..

[32] Chen J., Vistica B.P., Takase H., Ham D.I., Fariss R.N., Wawrousek E.F., Chan C.-C., DeMartino J.A., Farber J.M., Gery I. (2004). A unique pattern of up- and down-regulation of chemokine receptor CXCR3 on inflammation-inducing Th1 cells. Eur. J. Immunol..

[33] Wang W., Chong W.P., Li C., Chen Z., Chen J. (2019). Type I Interferon Therapy Limits CNS Autoimmunity by Inhibiting CXCR3-Mediated Trafficking of Pathogenic Effector T Cells. Cell Rep..

[34] Griseri T., Arnold I., Pearson C., Krausgruber T., Schiering C., Franchini F., Schulthess J., McKenzie B.S., Crocker P.R., Powrie F. (2015). Granulocyte Macrophage Colony-Stimulating Factor-Activated Eosinophils Promote Interleukin-23 Driven Chronic Colitis. Immunity.

[35] Astier A.L., Meiffren G., Freeman S., Hafler D.A. (2006). Alterations in CD46-mediated Tr1 regulatory T cells in patients with multiple sclerosis. J. Clin. Investig..

[36] El-Behi M., Ciric B., Dai H., Yan Y., Cullimore M., Safavi F., Zhang G.X., Dittel B.N., Rostami A. (2011). The encephalitogenicity of T(H)17 cells is dependent on IL-1- and IL-23-induced production of the cytokine GM-CSF. Nat. Immunol..

[37] Vignali D.A.A., Kuchroo V.K. (2012). IL-12 family cytokines: Immunological playmakers. Nat. Immunol..

[38] Fitzgerald D.C., Zhang G.-X., El-Behi M., Fonseca-Kelly Z., Li H., Yu S., Saris C.J.M., Gran B., Ciric B., Rostami A. (2007). Suppression of autoimmune inflammation of the central nervous system by interleukin 10 secreted by interleukin 27–stimulated T cells. Nat. Immunol..

[39] Awasthi A., Carrier Y., Peron J.P.S., Bettelli E., Kamanaka M., Flavell R.A., Kuchroo V.K., Oukka M., Weiner H.L. (2007). A dominant function for interleukin 27 in generating interleukin 10–producing anti-inflammatory T cells. Nat. Immunol..

[40] Maynard C.L., Harrington L.E., Janowski K.M., Oliver J.R., Zindl C.L., Rudensky A.Y., Weaver C.T. (2007). Regulatory T cells expressing interleukin 10 develop from Foxp3+ and Foxp3- precursor cells in the absence of interleukin 10. Nat. Immunol..

[41] Triebel F. (1990). LAG-3, a novel lymphocyte activation gene closely related to CD4. J. Exp. Med..

[42] Charbonnier L.M., Van Duivenvoorde L.M., Apparailly F., Cantos C., Han W.G.H., Noel D., Duperray C., Huizinga T.W.J., Toes R.E.M., Jorgensen C. (2006). Immature Dendritic Cells Suppress Collagen-Induced Arthritis by In Vivo Expansion of CD49b+ Regulatory T Cells. J. Immunol..

[43] Apetoh L., Quintana F.J., Pot C., Joller N., Xiao S., Kumar D., Burns E.J., Sherr D.H., Weiner H.L., Kuchroo V.K. (2010). The aryl hydrocarbon receptor interacts with c-Maf to promote the differentiation of type 1 regulatory T cells induced by IL-27. Nat. Immunol..

[44] Silver P.B., Horai R., Chen J., Jittayasothorn Y., Chan C.-C., Villasmil R., Kesen M.R., Caspi R.R. (2015). Retina-specific T regulatory cells bring about resolution and maintain remission of autoimmune uveitis. J. Immunol..

[45] Do J., Kim D., Kim S., Valentin-Torres A., Dvorina N., Jang E., Nagarajavel V., DeSilva T.M., Li X., Ting A.H. (2017). Treg-specific IL-27Rα deletion uncovers a key role for IL-27 in Treg function to control autoimmunity. Proc. Natl. Acad. Sci. USA.

[46] Batten M., Li J., Yi S., Kljavin N.M., Danilenko D.M., Lucas S., Lee J., de Sauvage F.J., Ghilardi N. (2006). Interleukin 27 limits autoimmune encephalomyelitis by suppressing the development of interleukin 17–producing T cells. Nat. Immunol..

[47] Stumhofer J.S., Laurence A., Wilson E.H., Huang E., Tato C.M., Johnson L.M., Villarino A.V., Huang Q., Yoshimura A., Sehy D. (2006). Interleukin 27 negatively regulates the development of interleukin 17-producing T helper cells during chronic inflammation of the central nervous system. Nat. Immunol..

[48] Fitzgerald D.C., Ciric B., Touil T., Harle H., Grammatikopolou J., Das Sarma J., Gran B., Zhang G.X., Rostami A. (2007). Suppressive effect of IL-27 on encephalitogenic Th17 cells and the effector phase of experimental autoimmune encephalomyelitis. J. Immunol..

[49] Vistica B.P., Takase H., Chan C., Rittling S., Kaer L.V., Gery I. (2004). EAU Does Not Always Resemble Other Autoimmune Diseases. Investig. Ophthalmol. Vis. Sci..

[50] Haak S., Croxford A.L., Kreymborg K., Heppner F.L., Pouly S., Becher B., Waisman A. (2009). IL-17A and IL-17F do not contribute vitally to autoimmune neuro-inflammation in mice. J. Clin. Investig..

[51] Villarino A., Hibbert L., Lieberman L., Wilson E., Mak T., Yoshida H., Kastelein R.A., Saris C., Hunter C.A. (2003). The IL-27R (WSX-1) Is Required to Suppress T Cell Hyperactivity during Infection. Immunity.

[52] Villarino A.V., Stumhofer J.S., Saris C.J., Kastelein R.A., de Sauvage F.J., Hunter C.A. (2006). IL-27 limits IL-2 production during Th1 differentiation. J. Immunol..

[53] Yoshimura T., Takeda A., Hamano S., Miyazaki Y., Kinjyo I., Ishibashi T., Yoshimura A., Yoshida H. (2006). Two-sided roles of IL-27: Induction of Th1 differentiation on naive CD4+ T cells versus suppression of proinflammatory cytokine production including IL-23-induced IL-17 on activated CD4+ T cells partially through STAT3-dependent mechanism. J. Immunol..

[54] Damsker J.M., Hansen A.M., Caspi R.R. (2010). Th1 and Th17 cells: Adversaries and collaborators. Ann. N. Y. Acad. Sci..

[55] Amadi-Obi A., Yu C.R., Liu X., Mahdi R.M., Clarke G.L., Nussenblatt R.B., Gery I., Lee Y.S., Egwuagu C.E. (2007). TH17 cells contribute to uveitis and scleritis and are expanded by IL-2 and inhibited by IL-27/STAT1. Nat. Med..

[56] Diveu C., McGeachy M.J., Boniface K., Stumhofer J.S., Sathe M., Joyce-Shaikh B., Chen Y., Tato C.M., McClanahan T.K., de Waal Malefyt R. (2009). IL-27 blocks RORc expression to inhibit lineage commitment of Th17 cells. J. Immunol..

[57] Sonoda K.H., Yoshimura T., Takeda A., Ishibashi T., Hamano S., Yoshida H. (2007). WSX-1 plays a significant role for the initiation of experimental autoimmune uveitis. Int. Immunol..

[58] Casella G., Finardi A., Descamps H., Colombo F., Maiorino C., Ruffini F., Patrone M., Degano M., Martino G., Muzio L. (2017). IL-27, but not IL-35, inhibits neuroinflammation through modulating GM-CSF expression. Sci. Rep..

[59] Zhu J., Liu J.-Q., Liu Z., Wu L., Shi M., Zhang J., Davis J.P., Bai X.-F. (2018). Interleukin-27 Gene Therapy Prevents the Development of Autoimmune Encephalomyelitis but Fails to Attenuate Established Inflammation due to the Expansion of CD11b(+)Gr-1(+) Myeloid Cells. Front. Immunol..

[60] Burmester G.R., Weinblatt M.E., McInnes I.B., Porter D., Barbarash O., Vatutin M., Szombati I., Esfandiari E., Sleeman M.A., Kane C.D. (2013). Efficacy and safety of mavrilimumab in subjects with rheumatoid arthritis. Ann. Rheum. Dis..

[61] McQualter J.L., Darwiche R., Ewing C., Onuki M., Kay T.W., Hamilton J.A., Reid H.H., Bernard C.C. (2001). Granulocyte macrophage colony-stimulating factor: A new putative therapeutic target in multiple sclerosis. J. Exp. Med..

[62] Constantinescu C.S., Asher A., Fryze W., Kozubski W., Wagner F., Aram J., Tanasescu R., Korolkiewicz R.P., Dirnberger-Hertweck M., Steidl S. (2015). Randomized phase 1b trial of MOR103, a human antibody to GM-CSF, in multiple sclerosis. Neurol. Neuroimmunol. Neuroinflamm..

[63] Sheng W., Yang F., Zhou Y., Yang H., Low P.Y., Kemeny D.M., Tan P., Moh A., Kaplan M.H., Zhang Y. (2014). STAT5 programs a distinct subset of GM-CSF-producing T helper cells that is essential for autoimmune neuroinflammation. Cell Res..

[64] Bing S.J., Silver P.B., Jittayasothorn Y., Mattapallil M.J., Chan C.-C., Horai R., Caspi R.R. (2020). Autoimmunity to neuroretina in the concurrent absence of IFN-γ and IL-17A is mediated by a GM-CSF-driven eosinophilic inflammation. J. Autoimmun..

[65] (1895). Cutting Edge: Suppression of GM-CSF Expression in Murine and Human T Cells by IL-27. J. Immunol..

[66] Lindner E., Steinwender G., Plainer S., Poeschl E.M., Weger M., Ardjomand N., Renner W., El-Shabrawi Y. (2013). Role of IL-10 gene polymorphisms in intermediate and HLA-B27-associated uveitis. Acta Ophthalmol..

[67] Agarwal R.K., Horai R., Viley A.M., Silver P.B., Grajewski R.S., Su S.B., Yazdani A.T., Zhu W., Kronenberg M., Murray P.J. (2008). Abrogation of anti-retinal autoimmunity in IL-10 transgenic mice due to reduced T cell priming and inhibition of disease effector mechanisms. J. Immunol..

[68] de Araujo E.F., Feriotti C., Galdino N.A.L., Preite N.W., Calich V.L.G., Loures F.V. (2017). The IDO-AhR Axis Controls Th17/Treg Immunity in a Pulmonary Model of Fungal Infection. Front. Immunol..

[69] Stumhofer J.S., Silver J.S., Laurence A., Porrett P.M., Harris T.H., Turka L.A., Ernst M., Saris C.J.M., O’Shea J.J., Hunter A. (2007). Interleukins 27 and 6 induce STAT3-mediated T cell production of interleukin 10. Nat. Immunol..

[70] Pot C., Jin H., Awasthi A., Liu S.M., Lai C.Y., Madan R., Sharpe A.H., Karp C.L., Miaw S.-C., Ho I.-C. (2009). Cutting edge: IL-27 induces the transcription factor c-Maf, cytokine IL-21, and the costimulatory receptor ICOS that coordinately act together to promote differentiation of IL-10-producing Tr1 cells. J. Immunol..

[71] Huang W., Solouki S., Carter C., Zheng S.G., August A. (2018). Beyond Type 1 Regulatory T Cells: Co-expression of LAG3 and CD49b in IL-10-Producing T Cell Lineages. Front. Immunol..

[72] Liu H., Rohowsky-Kochan C. (2011). Interleukin-27-Mediated Suppression of Human Th17 Cells Is Associated with Activation of STAT1 and Suppressor of Cytokine Signaling Protein 1. J. Interferon Cytokine Res..

[73] Murugaiyan G., Mittal A., Lopez-Diego R., Maier L.M., Anderson D.E., Weiner H.L. (2009). IL-27 is a key regulator of IL-10 and IL-17 production by human CD4+ T cells. J. Immunol..

[74] Yeung I.Y.L., Popp N.A., Chan C.-C. (2015). The role of sex in uveitis and ocular inflammation. Int. Ophthalmol. Clin..

[75] Conti P., Younes A. (2020). Coronavirus COV-19/SARS-CoV-2 affects women less than men: Clinical response to viral infection. J. Biol. Regul. Homeost. Agents.

[76] Horai R., Caspi R.R. (2011). Cytokines in autoimmune uveitis. J. Interferon Cytokine Res..

[77] Huang C.C., Schleisman M., Choi D., Mitchell C., Rosenbaum J.T. (2019). Preliminary Report on Interleukin-22, GM-CSF, and IL-17F in the Pathogenesis of Acute Anterior Uveitis. Ocul. Immunol. Inflamm..

