# Supplementary material for: Deficiency of IL-27 Signaling Exacerbates Experimental Autoimmune Uveitis with Elevated Uveitogenic Th1 and Th17 Responses
Source: Int J Mol Sci. 2021 Jul 14;22(14):7517. doi: 10.3390/ijms22147517 (PMC8305313; doi:10.3390/ijms22147517)
Supplement: Supplementary file 1 [file ijms-22-07517-s001.zip › ijms-1238052-supplementary.pdf]

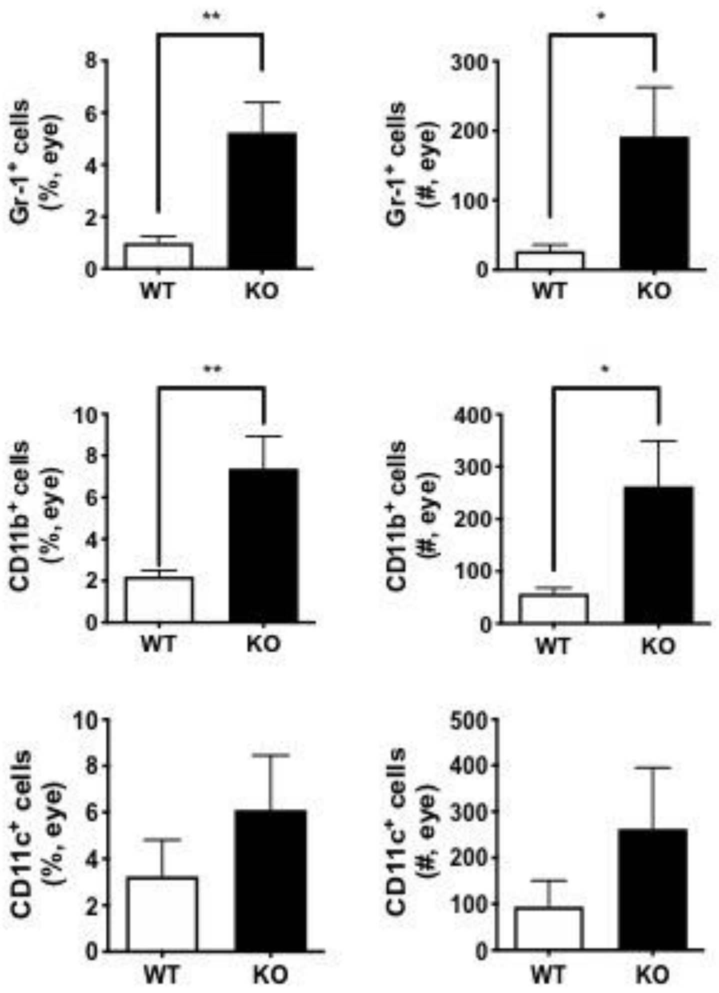

Supplementary Figure S1. Myeloid cells infiltration increased in inflamed eyes of IL-27Rα$^{-/-}$ (KO) EAU mice. Frequency and actual number of myeloid cells in the inflamed eyes from WT and KO EAU mice were determined by FCM analysis. The data represent the means ± SEMs from three independent experiments. * p <0.05, ** p <0.01, Mann-Whitney U test.

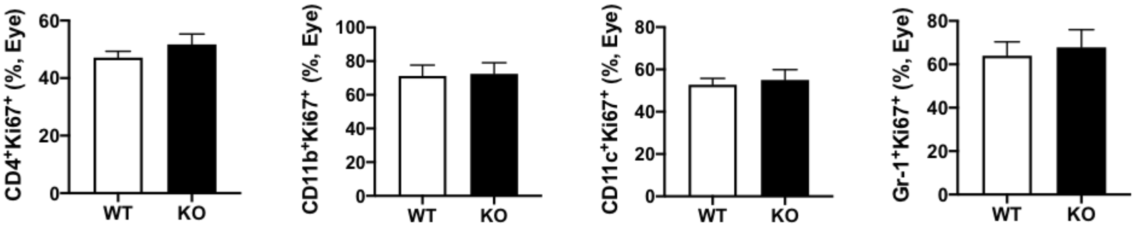

Supplementary Figure S2. Proliferation of ocular infiltrating CD4^{+} T cells and myeloid cells in EAU mice. Expression of Ki-67 by ocular infiltrating CD4^{+} T cells, CD11c^{+}, CD11b^{+} and Gr-1^{+} myeloid cells in WT and IL-27R$\alpha^{-/-}$ EAU mice. Data are depicted as the means ± SEMs (WT: n = 3 mice; KO: n = 3 mice), Mann-Whitney *U* test.

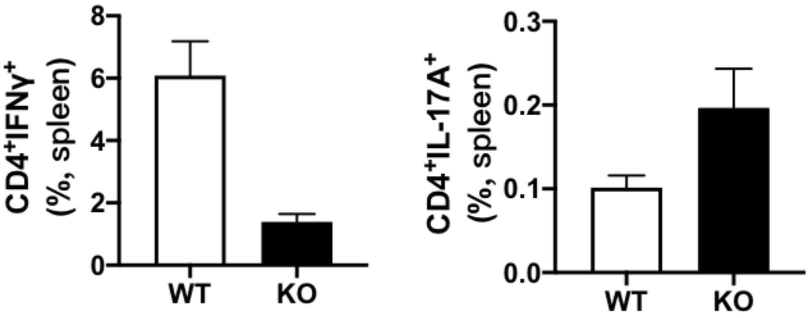

Supplementary Figure S3. Cytokine profile of splenic CD4$^+$ T cells from naïve IL-27Ra$^{-/-}$ (KO) mice and their WT littermates. IFN-γ and IL-17A production from splenic CD4$^+$ T cells were determined by FCM from naïve IL-27Rα$^{-/-}$ and WT littermates. The data represent the means ± SEMs (WT: n = 3 mice; KO: n = 3 mice), Mann-Whitney *U* test.

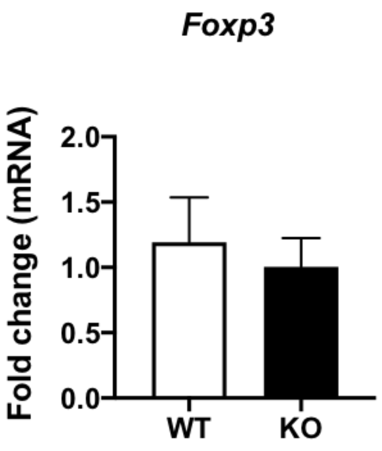

Supplementary Figure S4. Deficiency of IL-27Rα has no effect on *Foxp3* expression in CD4$^+$ T cells. The relative gene expression of *Foxp3* in CD4$^+$ T cells sorted from the spleens of WT and IL-27Rα$^{-/-}$ EAU mice. Total RNA was extracted from CD4$^+$ T cells that were sorted from the spleens of mice on day 21 post immunization. Data represent the means ± SEMs (4 mice per group), Mann-Whitney *U* test.
